# Crystal structures of heterotypic nucleosomes containing histones H2A.Z and H2A

**DOI:** 10.1098/rsob.160127

**Published:** 2016-06-29

**Authors:** Naoki Horikoshi, Yasuhiro Arimura, Hiroyuki Taguchi, Hitoshi Kurumizaka

**Affiliations:** 1 Research Institute for Science and Engineering, Waseda University, 2-2 Wakamatsu-cho, Shinjuku-ku, Tokyo 162-8480, Japan; 2 Laboratory of Structural Biology, Graduate School of Advanced Science and Engineering, Waseda University, 2-2 Wakamatsu-cho, Shinjuku-ku, Tokyo 162-8480, Japan; 3 Institute for Medical-oriented Structural Biology, Waseda University, 2-2 Wakamatsu-cho, Shinjuku-ku, Tokyo 162-8480, Japan

**Keywords:** H2A.Z, H2A.Z L1 loop, heterotypic nucleosome, nucleosome, chromatin, crystal structure

## Abstract

H2A.Z is incorporated into nucleosomes located around transcription start sites and functions as an epigenetic regulator for the transcription of certain genes. During transcriptional regulation, the heterotypic H2A.Z/H2A nucleosome containing one each of H2A.Z and H2A is formed. However, previous homotypic H2A.Z nucleosome structures suggested that the L1 loop region of H2A.Z would sterically clash with the corresponding region of canonical H2A in the heterotypic nucleosome. To resolve this issue, we determined the crystal structures of heterotypic H2A.Z/H2A nucleosomes. In the H2A.Z/H2A nucleosome structure, the H2A.Z L1 loop structure was drastically altered without any structural changes of the canonical H2A L1 loop, thus avoiding the steric clash. Unexpectedly, the heterotypic H2A.Z/H2A nucleosome is more stable than the homotypic H2A.Z nucleosome. These data suggested that the flexible character of the H2A.Z L1 loop plays an essential role in forming the stable heterotypic H2A.Z/H2A nucleosome.

## 1. Introduction

The nucleosome is a basic unit of eukaryotic chromatin, in which genomic DNA is compacted and accommodated within the nucleus. In the nucleosome, two copies each of histones H2A, H2B, H3 and H4 form the histone octamer, which wraps about 150 base-pairs of DNA on its surface [1]. Nucleosomes are connected with linker DNAs and form poly-nucleosomes. The local higher-order configurations of poly-nucleosomes are considered as determinants for the expression or repression of genes in certain loci [2,3].

The local higher-order chromatin configuration may be amended by various nucleosomes containing histone modifications and histone variants. Post-translational modifications of histones occur on specific chromosome loci and epigenetically regulate the gene expression of these loci through the higher-order chromatin configuration and dynamics [4–9]. Histone variants are also important epigenetic markers, which may dictate the functional regions of chromosomes or the specific loci of the genomic DNA [10–12]. Histone variants are encoded as non-allelic histone genes and have different amino acid sequences from those of the canonical histones [13].

Among the histone variants, H2A.Z is known as a universal nucleosome component and has been suggested to function as a regulator of transcription [14–16]. The contributions of H2A.Z in chromosome stability and DNA repair have also been reported [17–22]. H2A.Z is an essential factor for early development and stem cell differentiation in metazoans, but its role in these developmental stages remains poorly understood [23–27].

H2A.Z is known to accumulate around transcription start sites (TSSs), which frequently contain nucleosome-depleted regions (NDRs), especially in transcriptionally active genes [15,16,28–33]. Importantly, the depletion of H2A.Z reportedly enhances the nucleosomal barrier to RNA polymerase, suggesting that the H2A.Z nucleosome just downstream of the TSS (+1 nucleosome) may function to relieve the RNA polymerase pausing by the nucleosomal barrier at the TSS [34]. Therefore, the H2A.Z nucleosomes around TSSs may be required for transcriptional activation, due to their unstable character. Consistent with this idea, previous experiments designed to detect whole nucleosome disruption suggested that the H2A.Z nucleosome is less stable than the canonical nucleosome [35–37]. The instability of the H2A.Z nucleosome may be more significant, when the histone H3.3 variant is incorporated into the H2A.Z nucleosome [38]. However, a fluorescence resonance energy transfer assay, which specifically detects the H2A-H2B dissociation from the nucleosome, suggested that the dissociation of H2A.Z-H2B is more salt-resistant than that of H2A-H2B under high salt conditions (around 550 mM NaCl) [39].

The H2A.Z nucleosome (+1) of active genes reportedly shifts upstream and occupies the TSS regions during mitosis, when transcription is generally suppressed [40]. Intriguingly, in mouse trophoblast stem cells, the H2A.Z nucleosomes around TSS regions convert from homotypic (H2A.Z/H2A.Z) to heterotypic (H2A.Z/H2A) after DNA replication [41,42]. The heterotypic H2A.Z/H2A nucleosomes may occupy the TSS to regulate the transcription status of the related genes [41,42]. However, the previous crystal structures of the homotypic H2A.Z nucleosomes indicated that the L1 loop structure of H2A.Z is very different from that of canonical H2A and may cause steric hindrance when it forms the heterotypic nucleosome with canonical H2A [36,42,43].

To understand this intriguing discrepancy, in this study, we reconstituted the heterotypic H2A.Z/H2A nucleosomes and determined their crystal structures. The structures revealed that the H2A.Z L1 loop configuration drastically changes upon heterotypic nucleosome formation, without any structural change of the canonical H2A structure. To our surprise, we found that the heterotypic H2A.Z/H2A nucleosome is more stable than the homotypic H2A.Z nucleosome. These structural and biochemical properties of nucleosomes containing H2A.Z, homotypically and heterotypically, are important to understand the mechanism by which the H2A.Z-dependent transcriptional regulation is epigenetically maintained and promoted in cells.

## 2. Results and discussion

### 2.1. Preparation of the heterotypic H2A.Z/H2A nucleosome

**Figure 1.**
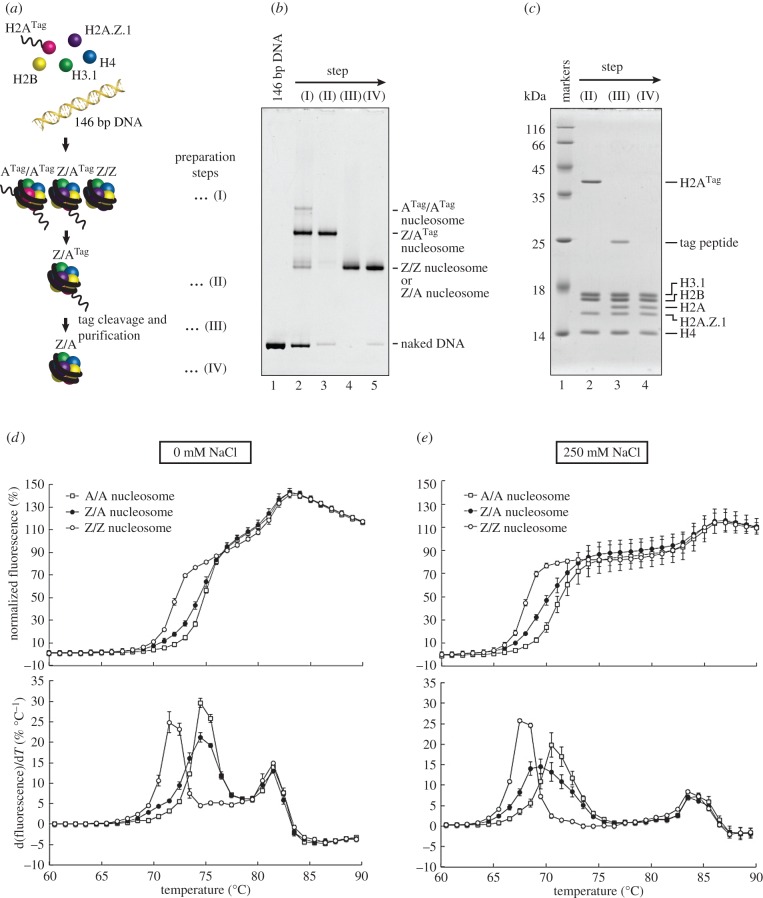
Preparation and thermal stability of the heterotypic H2A.Z/H2A nucleosome. (*a*) Schematic representation of the heterotypic H2A.Z/H2A nucleosome preparation. (I), (II), (III) and (IV) indicate each preparation step. The 144aa and His_6_ tagged H2A peptide are denoted as A^Tag^. (*b*) A native 6% polyacrylamide gel electrophoresis (PAGE) image of the heterotypic H2A.Z/H2A nucleosomes at each preparation step. Lanes 2–5 are the samples corresponding to preparation steps (I)–(IV), respectively. Lane 1 is the naked 146 base-pair DNA. The gel was stained with ethidium bromide. (*c*) An SDS-16% PAGE image of the heterotypic H2A.Z/H2A nucleosomes at each preparation step. Lanes 2–4 are the samples corresponding to preparation steps (II)–(IV), respectively. Lane 1 is molecular mass markers. The gel was stained with Coomassie Brilliant Blue. (*d*,*e*) Thermal stability assay for the heterotypic H2A.Z/H2A, homotypic H2A.Z and homotypic H2A nucleosomes. Thermal denaturation curves of the heterotypic H2A.Z/H2A nucleosome (black circles), the homotypic H2A.Z nucleosome (white circles) and the homotypic H2A nucleosome (grey squares) are presented. The fluorescence intensity at each temperature was normalized relative to that at 95°C, and the normalized values were plotted against temperatures ranging from 60°C to 90°C (upper panels). The derivative values of the thermal denaturation curves in the upper panels are plotted in the lower panels. Means ± s.d. (*n* = 3–4) are shown. (*d*) Experiments conducted in the absence of NaCl. (*e*) Experiments conducted in the presence of 250 mM NaCl.

To understand the mechanism by which the H2A.Z and H2A molecules are heterotypically accommodated within a nucleosome, we reconstituted the heterotypic H2A.Z/H2A nucleosome, with H3.1 as the histone H3 subunit, by a method based on previous studies [44,45] (figure 1*a*). In mammals, H2A.Z.1 and H2A.Z.2 are found as two non-allelic isoforms [46]. The H2A.Z.1 knockout in mice is lethal, indicating its essential role in development [24]. Therefore, in this study, we used H2A.Z.1 as the representative H2A.Z.

Human histone H2A was prepared as a fusion protein containing an additional 144 amino acid peptide (Tag) at its N-terminus, just before the thrombin recognition sequence, Leu-Val-Pro-Arg-Gly-Ser (H2A^Tag^; electronic supplementary material, table S1). The nucleosomes were reconstituted by the salt-dialysis method with H2A.Z and H2A^Tag^, in the presence of H2B, H3.1 and H4. In this step, the homotypic H2A^Tag^ nucleosome, the homotypic H2A.Z nucleosome and the heterotypic H2A.Z/H2A^Tag^ nucleosome were reconstituted (figure 1*b*, lane 2). These three nucleosomes were separated very well by native polyacrylamide gel electrophoresis (PAGE) (figure 1*b*, lane 2). We then purified the heterotypic nucleosome by preparative native PAGE (figure 1*b*, lane 3 and figure 1*c*, lane 2). The tag peptide was proteolytically removed from the H2A portion (figure 1*b*, lane 4 and figure 1*c*, lane 3) and the heterotypic H2A.Z/H2A nucleosome was further purified by preparative native PAGE (figure 1*b*, lane 5). The purified heterotypic H2A.Z/H2A nucleosome contained both H2A.Z and H2A, in addition to H2B, H3.1 and H4 (figure 1*c*, lane 4).

### 2.2. Stability of the heterotypic H2A.Z/H2A nucleosome

We then performed a thermal stability assay to evaluate the stabilities of the reconstituted nucleosomes [45,47,48]. In this method, the histones thermally dissociated from the nucleosome are detected by the fluorescent signal of SYPRO Orange bound to free histones. Consistent with the previous results [48], the canonical H2A nucleosome showed a bi-phasic thermal denaturation curve (figure 1*d*, upper panel) with two dissociation temperature peaks at 74–75°C and 81–82°C, corresponding to the H2A-H2B and H3-H4 dissociations, respectively, under the conditions without NaCl (figure 1*d*, lower panel). In the homotypic H2A.Z nucleosome, H2A.Z-H2B dissociated from the nucleosome at a lower temperature than H2A-H2B in the canonical H2A nucleosome (figure 1*d*). These results indicated that the homotypic H2A.Z nucleosome is less stable than the canonical H2A nucleosome. To our surprise, the heterotypic H2A.Z/H2A nucleosome was clearly more stable than the homotypic H2A.Z nucleosome, but slightly less stable than the canonical H2A nucleosome (figure 1*d*). This moderate stability of the heterotypic H2A.Z/H2A nucleosome was also confirmed under the conditions with 250 mM NaCl (figure 1*e*). Therefore, the presence of canonical H2A may facilitate the H2A.Z-H2B association with the H3-H4 tetramer and/or DNA in the nucleosome.

### 2.3. The H2A.Z L1 loop structure is drastically altered in the heterotypic H2A.Z/H2A nucleosome

**Figure 2.**
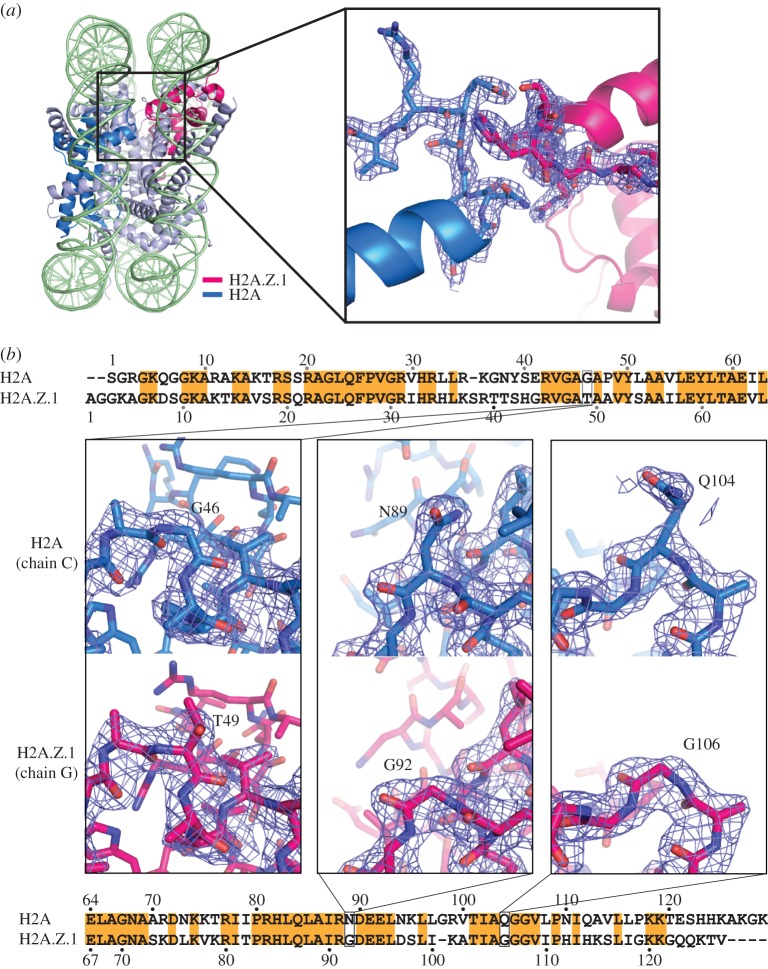
Crystal structure of the heterotypic H2A.Z/H2A nucleosome. (*a*) Overall structure of the heterotypic H2A.Z/H2A nucleosome with H3.1. The H2A.Z and H2A molecules are coloured pink and blue, respectively. The region around the L1 loops encircled by a rectangle is enlarged and presented in the right panel, with the 2mFo-DFc maps contoured at the 1.5*σ* level. (*b*) Close-up views of the H2A.Z- and H2A-specific residues. The H2A Gly46, Asn89 and Gln104 residues and the H2A.Z Thr49, Gly92 and Gly106 residues are presented. The 2mFo-DFc maps were calculated and contoured at the 1.5*σ* level. The human histone H2A and H2A.Z.1 sequences are aligned and the conserved residues are encircled by orange rectangles.

To reveal the structural basis for the heterotypic H2A.Z/H2A nucleosome formation and stability, we determined the crystal structure of the heterotypic H2A.Z/H2A nucleosome at 2.2 Å resolution (figure 2*a* and table 1). In the crystal structure, the electron densities for the H2A.Z-specific residues, such as Thr49, Gly92 and Gly106, are clearly distinguishable from the corresponding H2A-specific residues, Gly46, Asn89 and Gln104 (figure 2*b*). Previous structural studies suggested that the H2A.Z L1 loop region sterically clashes with the H2A L1 loop region in the heterotypic nucleosome [36,42,43]. Importantly, we found that the electron densities of the H2A.Z and H2A L1 loop regions were clearly visible, and thus the L1 loops are stably accommodated without steric clash between the H2A.Z and H2A molecules (figure 2*a*, right panel; electronic supplementary material, figure S1).

**Table 1.** Data collection and refinement statistics (molecular replacement).

	heterotypic H2A.Z/H2A nucleosome with H3.1	heterotypic H2A.Z/H2A nucleosome with H3.3	homotypic H2A.Z nucleosome with H3.3
data collection			
space group	P2_1_2_1_2_1_	P2_1_2_1_2_1_	P2_1_2_1_2_1_
cell dimensions			
*a*, *b*, *c* (Å)	105.1, 109.7, 181.5	98.2, 105.8, 166.3	104.8, 109.9, 181.9
α, β, γ (°)	90.0, 90.0, 90.0	90.0, 90.0, 90.0	90.0, 90.0, 90.0
resolution (Å)^a^	50–2.20 (2.28–2.20)	50–2.35(2.43–2.35)	50–2.92 (3.02–2.92)
*R*_sym_ or *R*_merge_^a^	9.3 (49.9)	10.0 (29.9)	7.0 (47.4)
*I*/*σI*^a^	12.7 (2.1)	8.3 (3.0)	14.1 (4.96)
completeness (%)^a^	97.9 (93.7)	99.4 (98.5)	99.7 (100)
redundancy^a^	4.6 (3.0)	7.4 (5.7)	6.8 (7.0)
refinement			
resolution (Å)^a^	48.62–2.20 (2.23–2.20)	49.10–2.35 (2.38–2.35)	39.01–2.92 (2.98–2.92)
no. reflections	104 756	72 359	45 868
* R*_work_/*R*_free_	22.54/27.07	22.66/25.93	20.52/25.21
no. atoms			
protein	5935	5940	5913
DNA	5980	5980	5980
ligand/ion	12	14	—
water	292	88	—
*B*-factors			
protein	40.08	38.61	55.25
DNA	90.99	68.63	110.92
ligand/ion	73.38	59.57	—
water	41.83	35.88	—
r.m.s. deviations			
bond lengths (Å)	0.010	0.003	0.011
bond angles (°)	1.121	0.579	1.202

^a^Values in parentheses are for highest-resolution shell.

**Figure 3.**
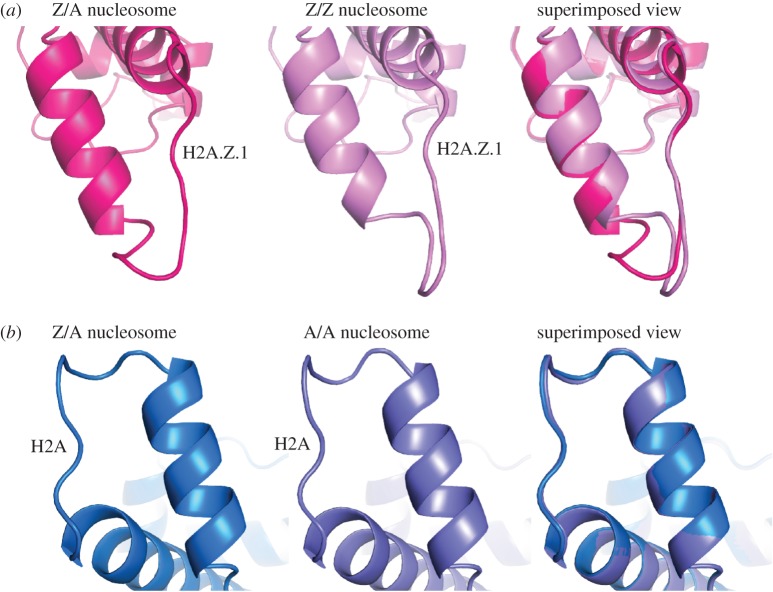
The H2A.Z L1 loop structure is drastically altered in the heterotypic H2A.Z/H2A nucleosome. (*a*) The H2A.Z L1 loop structures. The H2A.Z L1 loop structure in the heterotypic H2A.Z/H2A nucleosome (left panel) and that in the homotypic H2A.Z nucleosome (central panel, PDB ID: 3WA9) are superimposed and are presented in the right panel. The H2A.Z molecules in the heterotypic and homotypic nucleosomes are coloured pink and light pink, respectively. (*b*) The H2A L1 loop structures. The H2A L1 loop structure in the heterotypic H2A.Z/H2A nucleosome (left panel) and that in the homotypic H2A nucleosome (central panel, PDB ID: 3AFA) are superimposed and are presented in the right panel. The H2A molecules in the heterotypic and homotypic nucleosomes are coloured blue and indigo, respectively.

We then compared the L1 loop structures of H2A.Z and H2A in the heterotypic nucleosome with those in the homotypic nucleosomes. We found that the H2A.Z L1 loop structure is drastically altered in the heterotypic nucleosome, when compared with that in the homotypic H2A.Z nucleosome (figure 3*a*). In this H2A.Z Ll loop structure, the H2A.Z.1-specific Ser38 residue, which is replaced by Thr in H2A.Z.2, does not contact the other residues, and thus it may not affect the L1 loop structure in the heterotypic H2A.Z/H2A nucleosome if it is replaced by Thr. Surprisingly, in the heterotypic H2A.Z/H2A nucleosome, no obvious difference was observed in the H2A L1 loop structures between the heterotypic and homotypic H2A nucleosomes (figure 3*b*). These results indicated that the conformation of the H2A.Z L1 loop flexibly changes to fit the H2A L1 loop structure in the heterotypic H2A.Z/H2A nucleosome.

### 2.4. Interactions of the H2A.Z L1 loop residues with the H2A L1 loop and DNA in the heterotypic nucleosome

**Figure 4.**
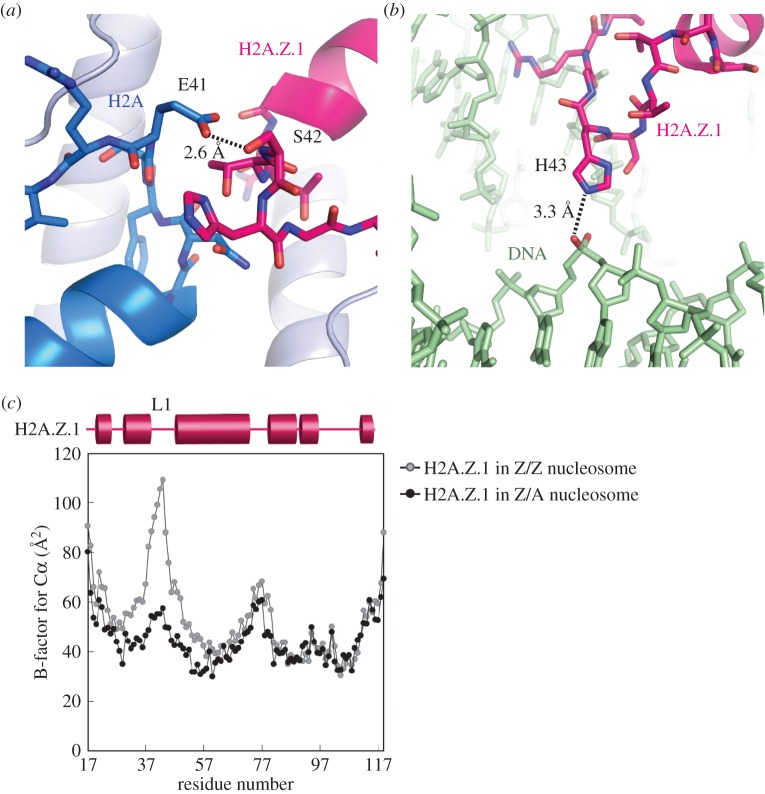
Interactions of the H2A.Z L1 loop residues with the H2A L1 loop and DNA in the heterotypic H2A.Z/H2A nucleosome. (*a*) Interaction between the H2A.Z Ser42 and H2A Glu41 residues in the heterotypic H2A.Z/H2A nucleosome. The dotted line indicates a possible hydrogen bond with a 2.6 Å length. The H2A.Z and H2A molecules are coloured pink and blue, respectively. (*b*) Interaction between H2A.Z His43 and a backbone phosphate of the DNA. The dotted line indicates a possible hydrogen bond with a 3.3 Å length. The H2A.Z and DNA molecules are coloured pink and bright green, respectively. (*c*) The B-factors for each C*α* atom of H2A.Z in the heterotypic H2A.Z/H2A nucleosome (black circles). As a reference, the B-factors for each Cα atom of H2A.Z in the homotypic H2A.Z nucleosome (grey circles, PDB ID: 3WA9) are also plotted. The secondary structure of H2A.Z in the nucleosomes is shown at the top of the panel.

In the heterotypic H2A.Z/H2A nucleosome, the H2A.Z Ser42 residue forms a hydrogen bond with the H2A Glu41 residue (figure 4*a*). In addition, the H2A.Z His43 residue forms a hydrogen bond with the DNA backbone in the heterotypic H2A.Z/H2A nucleosome (figure 4*b*). In the homotypic H2A.Z nucleosome, the B-factors for the Cα atoms of the H2A.Z L1 loops are extremely high, when compared with the other regions, indicating that the L1 loops are flexible (figure 4*c*). Interestingly, in the heterotypic H2A.Z/H2A nucleosome, the B-factors for the Cα atoms of the H2A.Z L1 loop were quite low (figure 4*c*). Similarly, the B-factors for the Cα atoms of the canonical H2A L1 loop were also low in the homotypic H2A nucleosome [36]. The specific H2A.Z L1 loop interactions with the H2A L1 loop and the DNA backbone may stabilize the H2A.Z L1 loop configuration in the heterotypic H2A.Z/H2A nucleosome, and thus potentially influence the nucleosome stability.

### 2.5. Incorporation of histone H3.3 does not affect the structure of the heterotypic H2A.Z/H2A nucleosome

**Figure 5.**
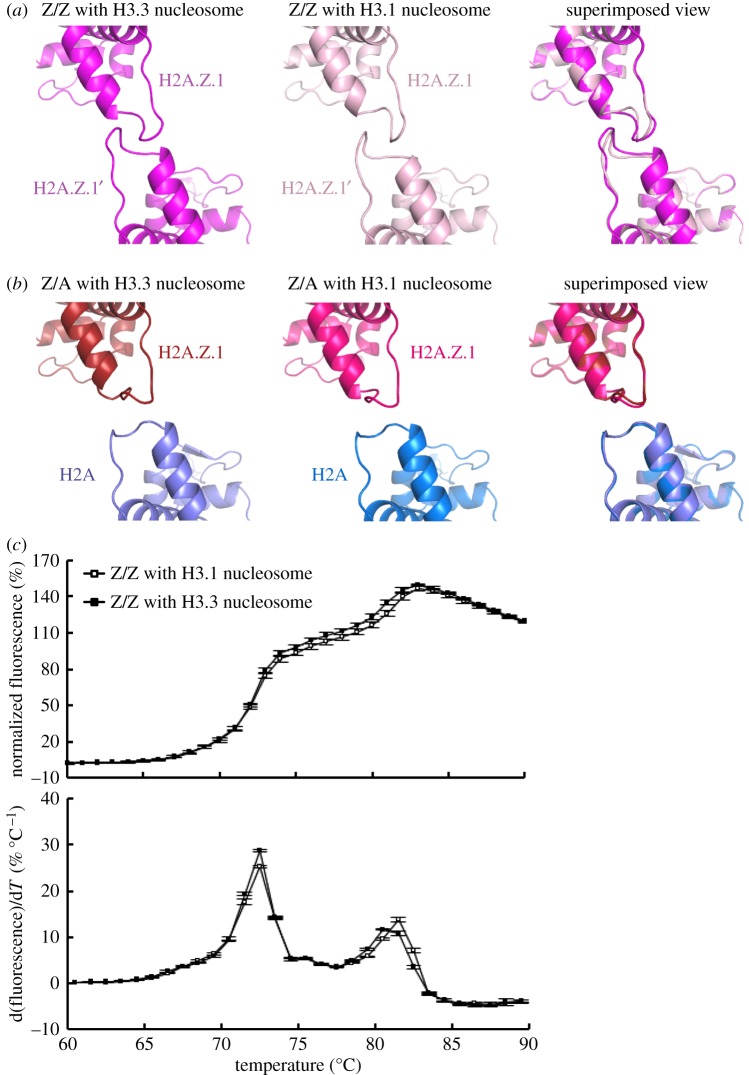
H3.3 does not affect the structure and stability of the heterotypic H2A.Z/H2A and homotypic H2A.Z nucleosomes. (*a*) The structure of the H2A.Z L1 loop regions in the homotypic H2A.Z nucleosome with H3.3 and the corresponding regions of that with H3.1 (PDB ID: 3WA9) are presented in the left and central panels, respectively. These H2A.Z L1 loop structures of the nucleosomes with H3.3 and H3.1 are superimposed and presented in the right panel. (*b*) The structure of the H2A.Z and H2A L1 loop regions in the heterotypic H2A.Z/H2A nucleosome with H3.3 and the corresponding regions of that with H3.1 are presented in the left and central panels, respectively. These H2A.Z L1 loop structures of the nucleosomes with H3.3 and H3.1 in the heterotypic H2A.Z/H2A nucleosomes are superimposed and presented in the right panel. (*c*) Thermal stability assay of the homotypic H2A.Z nucleosome with H3.3. Thermal denaturation curves of homotypic H2A.Z nucleosomes with H3.3 (black circles) and H3.1 (white circles) are presented. The experiments were performed in the absence of NaCl. The fluorescence intensity at each temperature was normalized relative to that at 95°C and the normalized values were plotted against temperatures ranging from 60°C to 90°C (upper panels). The derivative values of the thermal denaturation curves in the upper panels were plotted (lower panels). Means ± s.d. (*n* = 3) are shown.

As the H2A.Z nucleosome with a histone H3 variant, H3.3, at a TSS reportedly has different physical properties from the H2A.Z nucleosome with the canoncal H3.1 [38,49], we also tested whether the incorporation of H3.3 affects the structure of the H2A.Z molecule in nucleosomes. To do so, we crystallized the homotypic H2A.Z and heterotypic H2A.Z/H2A nucleosomes with H3.3 instead of H3.1, and determined their structures at 2.93 Å and 2.35 Å, respectively (table 1; electronic supplementary material, figures S2 and S3). We then found that the H3.3 incorporation minimally affected the H2A.Z L1 loop structures in both the homotypic H2A.Z and heterotypic H2A.Z/H2A nucleosomes (figure 5*a*,*b*). The stability of the homotypic H2A.Z nucleosome with H3.3 was the same as that with H3.1 (figure 5*c*). Therefore, we concluded that the H3.3 incorporation does not directly affect the structure and stability of the H2A.Z nucleosome. The instability found in the nucleosome containing H2A.Z and H3.3 may be induced by additional factors, such as histone chaperones, nucleosome remodellers, histone modifications and/or other nucleosome binding factors [20–22,27,30,32,33,37,50,51]

### 2.6. Perspective

In this study, we determined the crystal structures of the heterotypic H2A.Z/H2A nucleosomes. In chromatin, H2A.Z may function to preconfigure the poly-nucleosomes for removing and assembling transcription factors to *cis*-regulatory DNA elements [52]. This H2A.Z-mediated transcription regulation may play essential roles in various life phenomena, such as embryonic stem cell differentiation, temperature responses of genes and cognitive brain function [26,51,53,54]. Therefore, the structures and physical characteristics of the H2A.Z nucleosomes presented here provide an important structural basis for understanding how H2A.Z functions in complex life phenomena.

## 3. Material and methods

### 3.1. Preparation of recombinant human histones and histone complexes

The DNA fragment encoding the 144 amino acid (144aa) peptide tag sequence was inserted just upstream of the His_6_-tag sequence in the pET15b-H2A plasmid vector (electronic supplementary material, table S1). The 144aa tagged H2A peptide containing a His_6_-tag peptide was produced in *E. coli* BL21 (DE3) cells and was purified by Ni-NTA agarose (Qiagen) column chromatography. The 144aa and His_6_ tagged H2A peptide was dialysed against water four times and then lyophilized. H2A.Z.1, H2B, H3.1 (or H3.3) and H4 were purified as described previously [36,55,56]. The 144aa and His_6_ tagged H2A, H2A.Z.1, H2B, H3.1 (or H3.3) and H4 were mixed in 20 mM Tris-HCl buffer (pH 7.5), containing 7 M guanidine hydrochloride and 20 mM 2-mercaptoethanol, and the mixture was rotated at 4°C for 1.5 h. The sample was dialysed against 10 mM Tris-HCl buffer (pH 7.5), containing 2 M NaCl, 1 mM EDTA and 5 mM 2-mercaptoethanol. The resulting histone octamers containing one each of H2A.Z and tagged H2A (heterotypic), two tagged H2As (homotypic) and two H2A.Z.1 s (homotypic) were purified by HiLoad 16/60 Superdex200 (GE Healthcare) gel filtration column chromatography in 10 mM Tris-HCl buffer (pH 7.5), containing 2 M NaCl, 1 mM EDTA and 5 mM 2-mercaptoethanol.

### 3.2. Reconstitution and purification of the heterotypic H2A.Z/H2A nucleosomes

For the heterotypic H2A.Z/H2A nucleosome with H3.1, the mixture of the heterotypic H2A.Z/tagged H2A, homotypic tagged H2A and homotypic H2A.Z.1 octamers was mixed with the 146 base-pair human α-satellite derivative DNA, in a solution containing 2 M KCl. For the heterotypic H2A.Z/H2A nucleosome with H3.3, the tagged H2A-H2B dimer, H2A.Z.1-H2B dimer and H3.3-H4 tetramer were mixed with the 146 base-pair DNA, in a solution containing 2 M KCl. The KCl concentration was then gradually decreased to 0.25 M during dialysis. The resulting nucleosomes were then dialysed against 10 mM Tris-HCl buffer (pH 7.5), containing 0.25 M KCl, 1 mM EDTA and 1 mM dithiothreitol, at 4°C for 4 h. After this dialysis step, the samples were incubated at 55°C for 2 h to prevent improper histone–DNA binding. The heterotypic H2A.Z/tagged H2A nucleosome was purified by preparative native PAGE [45]. The tag peptide was proteolytically removed by thrombin protease (GE Healthcare) and the heterotypic H2A.Z/H2A nucleosome was further purified by another round of preparative native PAGE. For crystallization, the heterotypic H2A.Z/H2A nucleosome was dialysed against 20 mM potassium cacodylate buffer (pH 6.0), containing 1 mM EDTA.

### 3.3. Thermal stability assay of nucleosomes

The nucleosome stability was monitored by a thermal stability assay, as described previously [45,47,48]. Purified nucleosomes were mixed with SYPRO Orange dye (Sigma-Aldrich) in 20 mM Tris-HCl buffer (pH 7.5) containing 1 mM dithiothreitol, in the presence or absence of 250 mM NaCl. The SYPRO Orange fluorescence was detected with a StepOnePlus Real-Time PCR system (Applied Biosystems), using a temperature gradient from 25°C to 95°C, in steps of 1°C min^−1^.

### 3.4. Crystallization and structure determination

A 1 µl aliquot of each nucleosome solution (3 mg ml^−1^ for DNA concentration) was mixed with 1 µl of 20 mM potassium cacodylate buffer (pH 6.0), containing 50–70 mM KCl and 70–105 mM MnCl_2_. The mixture was equilibrated against 500 µl of reservoir solution, containing 20 mM potassium cacodylate (pH 6.0), 35–45 mM KCl and 45–60 mM MnCl_2_, by the hanging drop vapour diffusion method. Crystals of the nucleosomes were obtained by 2–3 weeks. The crystals were cryoprotected by soaking in reservoir solution additionally containing 28% (+/−)-2-methyl-2,4-pentanediol or 28% PEG400, and 2% trehalose, and were flash-cooled in a stream of N_2_ gas (−180°C). The diffraction datasets of the heterotypic H2A.Z/H2A nucleosomes with H3.1 or H3.3 were collected with an X-ray wavelength of 1.1 Å at −173°C on the BL-1A beamline at the Photon Factory (Tsukuba, Japan). The diffraction dataset of the homotypic H2A.Z nucleosomes with H3.3 was collected with an X-ray wavelength of 1.0 Å at −173°C on the BL41XU beamline at SPring-8. The datasets were processed using the HKL2000 and CCP4 programs [57,58]. The structures of the heterotypic H2A.Z/H2A nucleosomes with H3.1 or H3.3 were determined by molecular replacement with the PHASER program, using the crystal structure of the canonical nucleosome (PDB ID: 3AFA) as the search model [56,59]. The structure of the homotypic H2A.Z nucleosome with H3.3 was determined by molecular replacement with the PHASER program, using the crystal structure of the nucleosome containing H3.3 (PDB ID: 3AV2) as the search model [59,60]. The refinements and model building of the atomic coordinates were performed using the PHENIX and Coot programs [61,62]. The Ramachandran plot of the heterotypic H2A.Z/H2A nucleosome with H3.1 showed 98.1% of the residues in the favoured region, 1.9% of the residues in the allowed region and no outlying residues. The Ramachandran plot of the heterotypic H2A.Z/H2A nucleosome with H3.3 showed 98.1% of the residues in the favoured region, 1.9% of the residues in the allowed region and no outlying residues. The Ramachandran plot of the homotypic H2A.Z nucleosome with H3.3 showed 95.8% of the residues in the favoured region, 4.2% of the residues in the allowed region and no outlying residues. A summary of the data collection and refinement statistics is provided in table 1. Structural graphics were displayed using the PyMOL program (http://pymol.org).

### 3.5. B-factor calculation

The B-factors for the Cα atoms of the H2A.Z.1 molecules in the heterotypic H2A.Z/H2A nucleosome and the homotypic H2A.Z nucleosome (PDB ID: 3WA9) were calculated using the PHENIX program.

## Supplementary Material

Electronic supplementary material

## Data accessibility

The coordinates and structure factors of the crystal structures of the heterotypic H2A.Z/H2A nucleosome with H3.1, the heterotypic H2A.Z/H2A nucleosome with H3.3 and the homotypic H2A.Z.1 nucleosome with H3.3 have been deposited in the Protein Data Bank, under the accession codes 5B31, 5B32 and 5B33, respectively.
