# Supplementary material for: Crystal structures of heterotypic nucleosomes containing histones H2A.Z and H2A
Source: Open Biol. 2016 Jun 29;6(6):160127. doi: 10.1098/rsob.160127 (PMC4929947; doi:10.1098/rsob.160127)
Supplement: Electronic supplementary material [file rsob160127supp1.pdf]

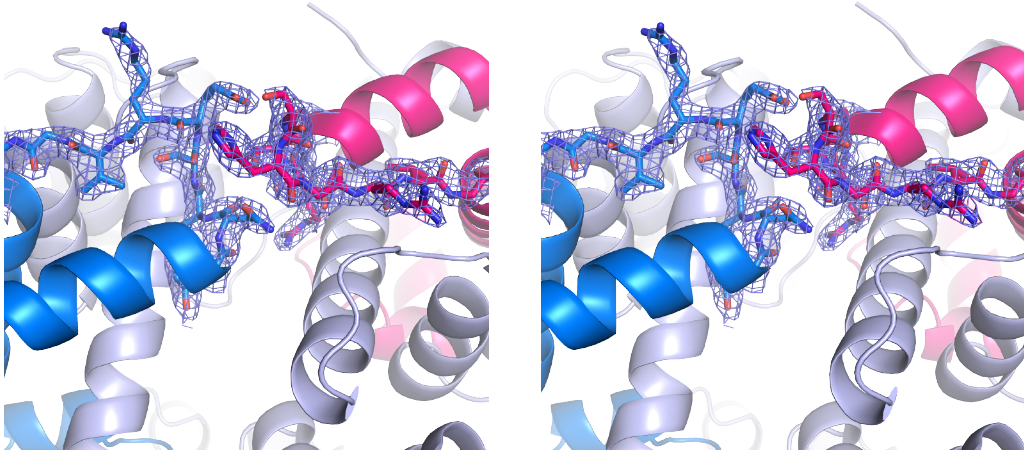

**Figure S1.** A stereoview of the H2A.Z and H2A L1 loop regions in the heterotypic H2A.Z/H2A nucleosome with H3.1. The H2A.Z and H2A molecules are colored pink and blue, respectively. The electron density map was calculated and contoured at the 1.5σ level.

(*a*)

(*b*)

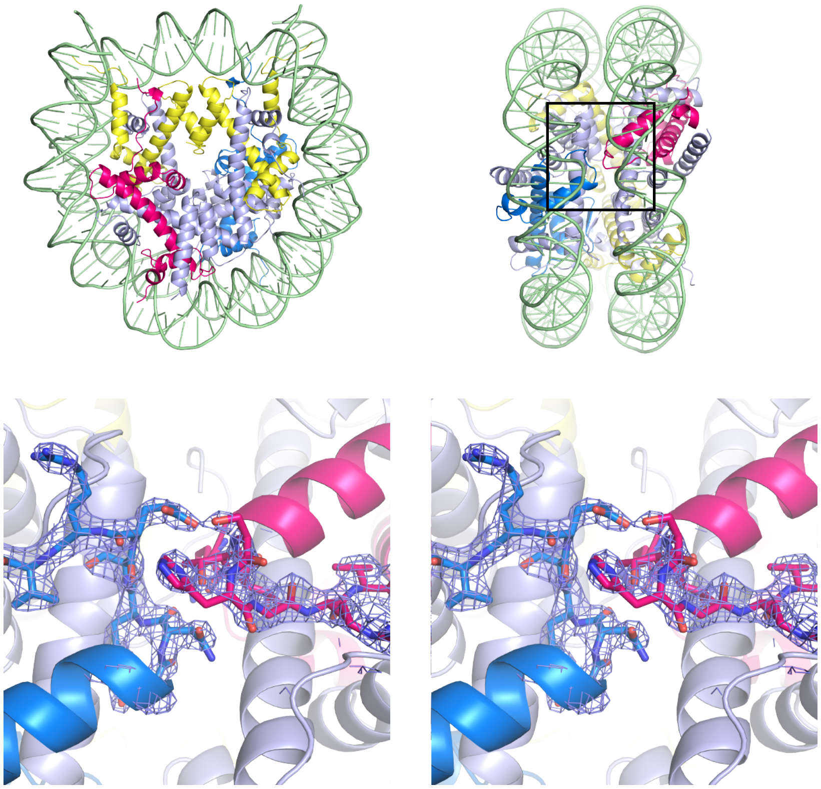

**Figure S2.** The crystal structure of the heterotypic H2A.Z/H2A nucleosome with H3.3. (*a*) Two views of the overall structure of the heterotypic H2A.Z/H2A nucleosome with H3.3 are presented. The H2A.Z, H2A, and H3.3 molecules are colored pink, blue, and yellow, respectively. The rectangle indicates the enlarged region presented in panel (*b*). (*b*) A stereoview of the H2A.Z and H2A L1 loop regions in the heterotypic H2A.Z/H2A nucleosome with H3.3. The H2A.Z and H2A molecules are colored pink and blue, respectively. The electron density map was calculated and contoured at the 1.5σ level.

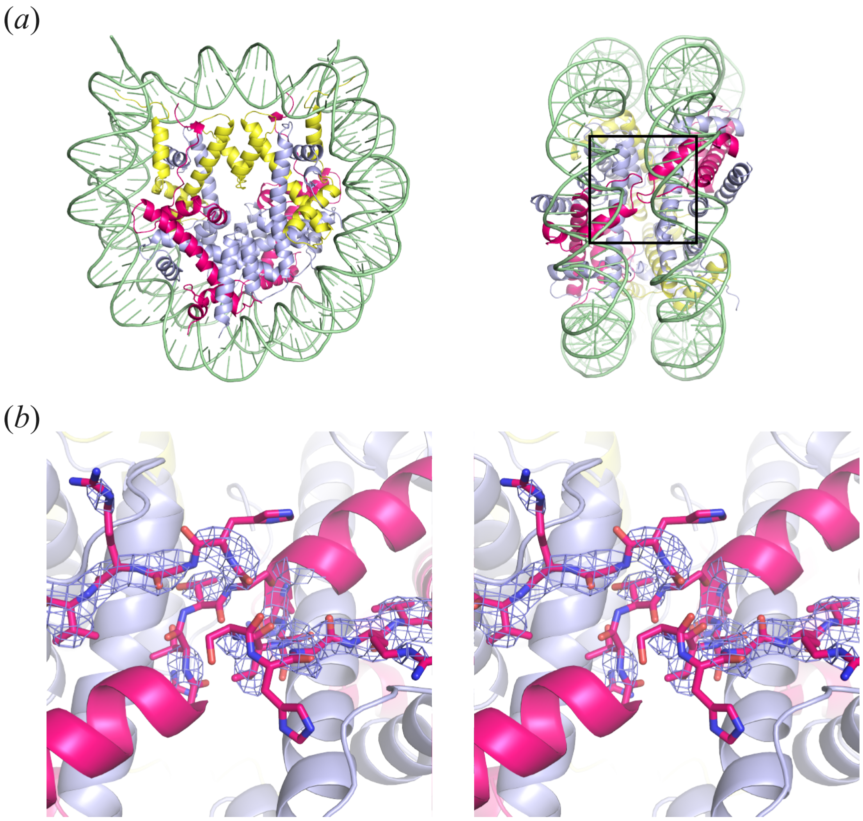


**Figure S3.** The crystal structure of the homotypic H2A.Z nucleosome with H3.3. (*a*) Two views of the overall structure of the homotypic H2A.Z nucleosome with H3.3 are presented. The H2A.Z and H3.3 molecules are colored pink and yellow, respectively. The rectangle indicates the enlarged region presented in panel (*b*). (*b*) A stereoview of the H2A.Z L1 loop regions in the homotypic H2A.Z nucleosome with H3.3. The H2A.Z molecules are colored pink. The electron density map was calculated and contoured at the 1.5σ level.

**Table S1.** The amino acid sequence of Tagged H2A.

**MGSSHHHHHHSSGMAKPAEEQLDVGQSKDENIHTSHITQDEFQRNSDRNM**

$His_6$ tag Tag

**EEHEEMGNDCVSKKQMPPVGSKKSSTRKDKEESKKKRFSSESKNKLVPEE**

**VTSTVTKSRRISRRPSDWWVVKSEETGSTTGSTGGSTGTTGSTGLVPRGS**

Thrombin recognition site

**HMSGRGKQGGKARAKAKTRSSRAGLQFPVGRVHRLLRKGNYSERVGAGAP**

H2A

**VYLAAVLEYLTAEILELAGNAARDNKKTRIIPRHLQLAIRNDEELNKLLG**

**RVTIAQGGVLPNIQAVLLPKKTESHHKAKGK**
